# Clinician Attitudes to Using Low-Dose Radiation Therapy to Treat COVID-19 Lung Disease

**DOI:** 10.1016/j.ijrobp.2020.12.003

**Published:** 2021-03-15

**Authors:** Catherine R. Hanna, Kathryn A. Robb, Kevin G. Blyth, Robert J. Jones, Anthony J. Chalmers

**Affiliations:** ∗ Institute of Cancer Sciences, University of Glasgow, Glasgow, Scotland; † CRUK Clinical Trials Unit, Glasgow, Scotland; ‡ Institute of Health and Wellbeing, University of Glasgow, Glasgow, Scotland

## Abstract

**Purpose:**

Current treatments for coronavirus disease 2019 (COVID-19) lung disease have limited efficacy. Low-dose radiation therapy (LDRT) has received both interest and criticism as a potential treatment for this condition. In this qualitative study we explored clinicians’ perspectives to identify barriers to testing LDRT in clinical trials and implementing it in clinical practice.

**Methods and Materials:**

Semistructured interviews were undertaken with 6 clinicians from 3 medical disciplines. Interviews were recorded, transcribed verbatim, and analyzed thematically, using a framework approach. Common themes regarding barriers to using LDRT for COVID-19 lung disease were identified from the data.

**Results:**

Three categories of barriers emerged: (1) the potential to do harm to the patient, including difficulty in predicting harm and lack of existing data to inform quantification of risks; (2) the feasibility of trialing this novel treatment strategy in the clinical setting, in particular trial design and recruitment, patient selection and buy-in from relevant clinician groups; and (3) the logistics of delivering the treatment, in particular risks of transmission to other patients and resources required for patient transfer.

**Conclusions:**

This study identified several barriers that may impede the evaluation and subsequent implementation of LDRT as a treatment for COVID-19 lung disease, from the perspectives of clinicians in 3 relevant specialties. By documenting and articulating these concerns, we hope to enhance discussion of why these barriers exist, and enable them to be addressed in a proactive manner to facilitate research into the potential benefits of radiation treatment for patients with COVID-19 lung disease going forward.

## Introduction

The severe acute respiratory syndrome coronavirus 2 (SARS-CoV-2) is having an unprecedented health and societal impact, with more than 54 million cases and 1.3 million deaths reported globally.^1^ Patients with SARS-CoV-2 who develop severe pneumonia and acute respiratory distress syndrome have the highest mortality rates.^2^ The lung pathology is characterized by diffuse alveolar damage suggestive of acute respiratory distress syndrome with alveolar infiltration by immune cells and proinflammatory cytokines.^3^, ^4^, ^5^ Research efforts to find effective treatments have included evaluation of low-dose radiation therapy (LDRT) with the rationale that LDRT has anti-inflammatory and immune-modulatory effects, which may counteract the pro-inflammatory state observed in SARS-CoV2–infected patients.^5^^,^^6^

Initiation of clinical trials to test LDRT has been slow, with preliminary results from 2 studies treating a total of 10 patients reported to date.^7^^,^^8^ Individuals and groups within the radiation oncology community have expressed concerns about the acceptability and validity of using radiation therapy in this context.^9^^,^^10^ Better understanding of clinicians’ concerns regarding the use of LDRT for coronavirus disease 2019 (COVID-19) lung disease would enable these issues to be addressed proactively, facilitating high-quality research in this field. In this study we aimed to identify and characterize these concerns by investigating multidisciplinary clinician perspectives on the topic.

## Methods

Semistructured interviews were undertaken by one researcher in July and August 2020. Potential participants were consultant-level medical professionals working in the UK National Health Service within the disciplines of radiation oncology, intensive care, or respiratory medicine. These specialties were chosen to represent clinicians involved in treating patients with COVID-19 lung disease and/or responsible for delivering radiation treatment. A convenience sample, identified by members of the research team, was initially approached. Initially, there were 2 replies out of 6 individuals approached. A snowballing technique, whereby clinicians who participated or declined were asked to identify alternative participants, was used. In this way, an additional 7 clinicians were approached and 4 of these agreed to participate. In total, 13 clinicians were invited and 6 agreed to be interviewed (3 men, 3 women) with representation from all 3 medical disciplines (2 of each). This study received ethical approval from the University of Glasgow Medical, Veterinary, and Life Sciences Ethics Committee (project number: 200190165) and was approved by the Greater Glasgow and Clyde Research and Innovation Department (ref: GN20HS293).

The interviews were recorded using video conferencing, and the audio recordings transcribed verbatim. Thematic analysis^11^ using a framework approach^12^ was undertaken by 2 researchers (CRH, KAR) who each coded all of the interviews. Codes and themes were developed deductively and iteratively refined after coding each interview via comparison of coding outcomes and discussion of discrepancies. Microsoft Excel 2016 was used for all analyses.

## Results

Three broad categories of barriers to using and testing LDRT for COVID-19 emerged from the interviews (Fig. E1 in Appendix E1): (1) potential to do harm; (2) feasibility of clinically trialing this novel treatment strategy (“trialability”); and (3) logistics of treatment delivery. The key opinions from each participant are summarized in Table 1, and direct quotations that align with the themes identified are provided in Appendix E2 (Table E1)

**Table 1:** Clinician attitudes on using LDRT for COVID-19 lung disease

	Radiation oncology 1	Radiation oncology 2	Respiratory 1	Respiratory 2	ICU 1	ICU 2
Harms						
Predicting harms	Low dose may still harm. Disagreement with targeting lungs. Radiation oncologists deliver treatment; therefore, responsible for any harm caused	Idiosyncratic lung damage from LDRT likely and unpredictable. Low dose unlikely to cause harm in majority of patients.	Concern: pneumonitis. Lungs are a reasonable target. Not doing harm is a bigger priority than knowing the treatment works. Harm may be caused by transfer.	Imaging of lungs before treatment is advised. Potential harms around transfer highlighted.	Potential harm from LDRT due to worsening hypoxia. Colleagues may have concerns about lung damage from LDRT.	Open to the concept that LDRT will reduce inflammation. Agrees with lungs as target. Concerns regarding transfer. Some reassurance from low dose.
Lack of evidence and previous experience	Not reassured by contemporary data due to low patient numbers.	Changing landscape regarding COVID-19 treatments. Even if small trials done, not sufficient patient numbers to reassure regarding harm.	Trial data would be important to provide reassurance about harms. There is a current lack of evidence about the longer-term effects of COVID-19 on the lungs.	Aware of recent trial evidence for testing LDRT for COVID-19 lung disease. Lack of personal experience with radiation therapy for nonmalignant condition. Lack of information about the longer-term effects of COVID-19.	Lack of personal experience with radiation therapy treatments.	Some reassurance regarding the low dose used after reviewing previous literature on using LDRT for pneumonia.
Trialability						
Trial design and patient recruitment	Difficult to measure benefit/harm when LDRT trialed in few patients. Patient numbers may not be sufficient for recruitment going forward.	Benefit to change practice not be picked up in a small cohort. A proportion of patients will get better with no intervention. A primary endpoint of whether patients get better would need large patient numbers to see a difference.	Distinguishing clinical signs and symptoms from COVID-19 versus treatment is challenging because of rapid deterioration in patients with COVID-19.	Reduction in oxygen requirement reasonable endpoint. Not clear at what time-point that end-point would be measured. Patients are very interested in participating in COVID-19 trials.	Uncertainty regarding COVID-19 patient numbers going forward. Treatment effect has to be large if numbers are small. Mortality-based end-point reasonable, mirroring the RECOVERY trial. Patients/families are very willing to participate in COVID-19 research.	Uncertainty regarding numbers of COVID-19 positive patients going forward. Recruiting large number to investigate clinically relevant treatment effect versus ability to deliver a trial needing high numbers. Patients may not like concept of LDRT but are generally engaged in COVID-19 research.
Clinician buy-in	COVID-19 trials easier to open due to clinical demand. Lack of buy-in from lung radiation oncologists. Physicians recruit but oncologists treat. Multidisciplinary approach	Clinician buy-in reduced due to the potential harm caused by LDRT.	Competition (for LDRT) with other novel treatments for COVID-19. Understanding how radiation therapy works in nonmalignant condition is important for buy-in.	Competition with other clinical trials testing novel treatments for COVID-19 is likely. Most clinicians see value in researching new treatments due to the lack of proven therapies.	Reduced clinician buy-in due to extra workload/fear of the unknown when it comes to radiation therapy.	Open to the idea of trialing LDRT and agreement with the potential biological plausibility.
Patient selection	Not doing harm vs risk in patients with no other options/those who recover spontaneously Avoid patients with unhealthy lungs who are very unwell. Obesity a risk factor and should be taken into account.	Avoid LDRT in young, fit patients who may recover spontaneously and those with lung fibrosis.	Obesity risk factor for poor outcomes for COVID-19 and should be considered. Avoid LDRT in early phase of the disease when patients are well as they may recover spontaneously.	Exclude underlying lung disease/acute conditions that worsen hypoxia (PE). Target those starting to deteriorate/ oxygen requirement/requiring level 2 care/d 10-14.	Avoid in patients on CPAP/very high levels of oxygen: logistics of transfer and treatment delivery difficult.	Avoid intubated patients/level 3 care. Those requiring level 2/HDU care reasonable. Many patients with COVID-19 lung disease will recover spontaneously.
Logistics						
Transfer	Staffing required for safe transfer to radiation therapy department is likely to be an issue.	Time spent in the radiation therapy department and potential for deterioration during transfer is likely to be an issue.	Logistics of treatment delivery is likely to be an issue.	Difficulty transporting unwell patients on oxygen. Staff required for transfer/ potential waiting time in the radiation therapy department.	Staffing required for transfer. Effect on other departments must be considered.	Transferring unwell patients and the effects on staffing. Compared with other COVID-19 trials, transfer is an extra issue.
Transmission	Staff/patients at risk of COVID-19 transmission. Transmission risk related to time spent by patients in radiation therapy department.	Logistics of separate rooms for COVID-19–positive patients/risk of transmission to others when COVID-19 positive patients are in transit.	Staff/patient transmission of COVID-19 is a concern. Cancer patients particularly vulnerable group in terms of infection risk.	Logistics of separating COVID-19–positive patients from non–COVID-19 patients.		Transmission risk during patient transfer from previous experience.
Resources for implementation				Implementation of LDRT would siphon resources away from other departments and patients.	Versus steroids, LDRT more difficult to scale up to population level. Potentially large pool of patients who are eligible if shown to be effective.	Resource implications for implementation of this treatment would be important.

*Abbreviations:* COVID-19 = coronavirus disease 2019; CPAP = continuous positive airway pressure; HDU = high dependency unit; ICU = intensive care unit; LDRT = low-dose radiation therapy; PE = pulmonary embolism.

### Harms

There was concern that LDRT could make an inflammatory state worse and that predicting the likelihood of this harm would be difficult. Radiation oncology participants in particular were concerned that it would not be possible to test patients’ lung function accurately, which is normal practice before prescribing radiation therapy for lung cancer. Some interviewees were reassured by the low radiation dose being used; others thought that the risk of exacerbating an inflammatory state was likely to be idiosyncratic and not dose dependent. Opinions were mixed regarding whether the lungs were the optimal target. One intensive care unit and one respiratory consultant agreed with treating the lungs whereas one of the radiation oncologists favored targeting lymphoid organs. There was a consistent opinion that assessing the risks and benefits was especially difficult because of the novelty of the treatment and the apparent lack of supporting data. It was highlighted that previous reports of using LDRT to treat pneumonia, both contemporary and historical, did not fully allay concerns about potential harms.

### Trialability

Participants discussed the challenge of designing a clinical trial with meaningful endpoints to measure treatment benefit, and of obtaining an adequate sample size. The primary endpoints suggested by these participants included mortality, specifically in line with the RECOVERY trial endpoint, as well as an endpoint that would measure a reduction in oxygen requirement. Several clinicians were of the opinion that the benefit would need to be large for 2 reasons: to be able to detect a benefit within a clinical trial (especially if patient numbers were small), and to support a change in practice. Participants were aware of preliminary data testing LDRT for COVID-19 lung disease in a small number of patients. Nevertheless, they noted that early-phase trials may not be large enough to provide reassurance regarding potential harms, and that proving efficacy of LDRT compared with standard care would be difficult because a proportion of patients would recover spontaneously. Difficulties in predicting future prevalence of COVID-19 and how this might affect recruitment to a clinical trial testing LDRT were also highlighted. Despite this, there was general agreement that it was easier to initiate and recruit to COVID-19 trials compared with trials in other disease types.

Second, the challenge of ensuring “buy-in” from clinicians was noted, especially given the novelty of the intervention and the perceived comparison to drug treatments being tested in competing trials. It was highlighted that the medical professional recruiting patients would likely be a respiratory physician and/or intensivist who is not familiar with radiation therapy, whereas the clinician delivering the treatment would be a radiation oncologist. This was considered unusual for a clinical trial and posed challenges regarding multidisciplinary working, communication, and trust.

Finally, the challenge of understanding which patients would or would not be optimal candidates for a trial was identified. No specific age cutoff was identified, and although obesity and ethnicity were mentioned as important patient-related risk factors, there was no consensus that these would be sufficient for patient selection. One of the respiratory physicians was aware of preliminary data from the RESCUE 1-19 trial, which recruited mainly older patients and expressed reassurance that including older patients in future trials would be acceptable.

There was agreement about excluding patients with underlying lung disease, additional reasons for hypoxemia such as pulmonary embolism, or requiring intensive care level medical support. These were all identified as factors that would increase the likelihood of harm as well as difficulties delivering treatment. Considering this dilemma, some clinicians suggested that optimal candidates might be those requiring level 2 hospital care, with a moderate oxygen requirement on approximately day 10 of the disease trajectory.

### Logistics

The final barriers that emerged were logistical. A major focus was the transfer of patients from medical wards to a radiation therapy department and, aside from the potential harm caused, the practicalities of performing that transfer within current resource levels. Participant comments related to the lack of higher level (intensive care unit) support on radiation therapy sites and the risk of transmission to other patients in the radiation therapy department. Lastly, the ability to implement LDRT in a real-world setting in terms of resources, cost, and scalability was seen as a barrier, even if it were shown to be effective in clinical trials.

## Discussion

This is the first study to capture clinician attitudes regarding the use of LDRT for COVID-19 lung disease. Our findings have implications for researchers evaluating the role of LDRT in COVID-19 lung disease and physicians considering its implementation in clinical practice.

Specifically, there was apprehension about aggravating pulmonary inflammation and a lack of evidence to inform the likelihood of this occurring. Emerging safety data from ongoing early-phase trials and appropriate patient selection, informed by knowledge of previous lung pathology and/or radiologic evaluation of baseline lung damage, will help to address this concern. Increasing understanding of the COVID-19 disease trajectory and data from control arms of ongoing randomized trials will also support identification of meaningful endpoints for larger-scale clinical trials. The concern regarding the distinction between the recruiting versus treating clinician raises an important point about the uniqueness of using radiation treatment for a nonmalignant condition and indicates that robust interdisciplinary relationships will be required for successful setup and running of a LDRT/COVID-19 trial. Finally, logistical concerns would need to be proactively addressed. The risk of transmission is not unique to radiation therapy, but there are specific challenges associated with vulnerable cancer patients receiving treatment in the same departments. Also, radiation therapy infrastructure is well developed in many countries but not always colocated with acute or intensive care facilities and capacity to treat additional, noncancer patients may be limited.

Participants in this study suggested that appropriate primary endpoints for a trial to investigate LDRT for COVID-19 lung disease would be mortality or a reduction in oxygen requirement. Future research is required to investigate what magnitude of benefit would need to be demonstrated in a clinical trial to support implementation of LDRT in a real-world setting. Our study participants made clear that any trial conducted to investigate LDRT may compete with trials assessing drug treatments, and that patients receiving LDRT in the context of a clinical trial would very likely already be receiving steroids, given the results of the RECOVERY trial.^13^ It would be particularly interesting to investigate how clinicians envisage LDRT would be used alongside other potential therapies such as steroids and antivirals, and if the benefit from LDRT would need to be at least equivalent to benefits seen for other novel therapies to be used in practice.

Our study has limitations. First, study participants were all from one location and it is unclear if the same barriers exist globally. If they do, it would be informative to understand how they were addressed and overcome in those centers in which trials have opened.^14^ This raises a broader question about variation in risk attitudes in the context of novel therapies between health care systems or geographic regions. We were not surprised that results from current trials were not commonly discussed, considering the findings from only one pilot study (n = 5) were published at the time the interviews were conducted.

Second, only 2 clinicians were interviewed from each discipline. The notion of sample size and data saturation within qualitative research remains much debated.^15^ The study was not intended to be a comprehensive investigation of all possible attitudes to LDRT for COVID-19 lung disease, but rather a pragmatic, yet informative and timely approach to explore common themes from interviewing a purposive sample of clinicians. Lastly, we did not seek the patient perspective on using LDRT for COVID-19 lung disease but appreciate this to be an important, complementary avenue of research.

In conclusion, we recognized the difficulty of setting up a multidisciplinary study investigating a novel therapy within the field of radiation oncology and have demonstrated the value of conducting a qualitative study to determine the feasibility and acceptability of this research. Specifically, we have identified the main barriers to the use of LDRT for COVID-19. Open discussion of these barriers will facilitate high-quality research in this field. Future research could build on the results from this study by using a more quantitative approach to investigate the magnitude of benefit that would be required from a clinical trial investigating LDRT for COVID-19 to change clinical practice.

## Supplementary Materials

Appendix E1

Appendix E2
