# Supplementary material for: Clinician Attitudes to Using Low-Dose Radiation Therapy to Treat COVID-19 Lung Disease
Source: Int J Radiat Oncol Biol Phys. 2021 Mar 15;109(4):886–90. doi: 10.1016/j.ijrobp.2020.12.003 (PMC7726525; doi:10.1016/j.ijrobp.2020.12.003)
Supplement: Appendix E1 [file mmc1.docx]

Figure E1: Themes identified from clinician interviews.

#
